# Atlanta Medical College

**Published:** 1878-10

**Authors:** 


ATLANTA MEDICAL COLLEGE.—The opening of this Institution was had on the 15th inst. by the introductory of Prof. Banks, in which sound, practical precepts were laid before the class.

The session commences with an unusually large attendance, and with the manifestation of more than usual zeal on the part of professors and students.

The stringency in financial matters and great distress in neighboring Western States from the scourge, were expected to interfere with an early attendance, and has no doubt delayed some who will yet swell the class.

We have not heard from other Medical Colleges, but hope cool weather will soon restore health to the cities which have so badly suffered, and that the Colleges at those places will soon be opened for teaching.

